# The Beneficial Role of Nrf2 in the Endothelial Dysfunction of Atherosclerosis

**DOI:** 10.1155/2022/4287711

**Published:** 2022-05-12

**Authors:** Zixia Huang, Mingyue Wu, Lijin Zeng, Deming Wang

**Affiliations:** ^1^Department of Anesthesiology, The Second Affiliated Hospital, Hengyang Medical School, University of South China, Hengyang, Hunan 421001, China; ^2^Department of Emergency, The First Affiliated Hospital, Sun Yat-Sen University, Guangzhou 510080, China

## Abstract

Cardiovascular disease (CVD) is a serious public health issue in China, accounting for more than 40% of all mortality, and it is the leading cause of death worldwide. Atherosclerosis is the pathological basis for much CVD, including coronary heart disease, acute myocardial infarction, and stroke. Endothelial dysfunction is an initiating and exacerbating factor in atherosclerosis. Recent research has linked oxidative stress and mitochondrial damage to endothelial dysfunction. Nuclear factor erythroid 2-related factor 2 (Nrf2) is a transcription factor with antioxidant effects that is strongly connected to several CVDs. However, the mechanism by which Nrf2 reduces CVD is unknown. Research indicates that Nrf2 improves endothelial function by resisting oxidative stress and mitochondrial damage, thereby delaying atherosclerosis. This article examines the mechanisms and potential targets of Nrf2 affecting endothelial cell function to improve atherosclerosis and to provide ideas for the development of new CVD treatments.

## 1. Introduction

Cardiovascular disease (CVD) is the leading cause of death and premature death in China, posing a significant public health risk. In 2019, CVD caused 18.6 million deaths worldwide and roughly 58% of all cardiovascular deaths in Asia [1]. The morbidity and mortality of CVD will only increase with the increasing elderly population [2]. At present, the treatment of CVD relies mainly on coronary revascularization and oral antiplatelet aggregation drugs. Coronary revascularization is an invasive operation with a risk of surgical complications, while long-term antiplatelet aggregation drug use has a risk of bleeding. Because many people cannot tolerate surgery or long-term antiplatelet drug therapy [3–5], clinicians hope to develop new methods for preventing and treating CVD.

Nuclear factor erythroid 2-related factor 2 (Nrf2) is a transcription factor with antioxidant effects strongly related to several CVDs [6]. Nrf2 regulates the biosynthesis, utilization, and regeneration of glutathione, thioredoxin, and NADPH, as well as the production of reactive oxygen species via the mitochondria and NADPH oxidase, to maintain cellular redox homeostasis [7]. Nrf2 can lower the risk of atherosclerosis-related chronic diseases by improving endothelial function [8–10]. This article examines the mechanisms and potential targets of Nrf2 affecting endothelial cell dysfunction and delaying atherosclerosis, starting from the pathophysiological basis of CVDs to provide ideas for the development of new therapeutic methods for CVDs.

## 2. Endothelial Cells

The blood vessel wall forms a selective barrier to molecular transport between blood and tissue, and endothelial cells form the inner lining of blood vessels, controlling the exchange of substances between blood and tissues. Endothelial cells form continuous thin monolayers that maintain vascular homeostasis by interacting with cells in the vessel wall and lumen [11]. They control vascular tone by releasing vasodilatory factors, such as nitric oxide (NO) and contractile factors; regulate blood flow and coagulation by producing factors that regulate platelet activity, the coagulation cascade, and the fibrinolytic system; and secrete adhesion molecules and cellular cytokines to coordinate the inflammatory response [12].

## 3. Endothelial Cells and Atherosclerosis

Atherosclerosis is a chronic disease process involving lipid accumulation [13]. It begins with endothelial cell dysfunction and is arbitrated by a cascade of intracellular and intercellular responses [14]. Endothelial cell dysfunction results in the infiltration of low-density lipoprotein (LDL) particles and their subsequent oxidation to oxidized LDL (oxLDL) [15]. Increased chemokine secretion by endothelial cells and increased adhesion protein expression on their surface allow them to recruit inflammatory cells, particularly monocytes, to the arterial intima. Monocytes differentiate into macrophages, which subsequently phagocytose lipids into foam cells, which undergo necrosis and apoptosis, forming the lipid core of progressive atherosclerotic lesions [16]. Injured endothelial cells secrete growth factors to activate smooth muscle cells (SMCs) in the arterial media, migrate into the intima through fenestrations in the inner elastic membrane, and phagocytose lipids mediated by lipoprotein lipase receptors on the surface to form SMC-derived foam cells. In the late stages of atherosclerosis, SMCs secrete extracellular matrix (collagen and elastin), forming fibrous caps that increase the instability of atherosclerotic plaques [17]. Communication between endothelial cells and other vascular cell populations in this atherogenic environment stimulates the release of proinflammatory cues, increasing the native inflammatory response and promoting atheromatous plaque development [18]. Reduced collagen synthesis and increased degradation due to inflammation cause progressive thinning of the fibrous cap, resulting in plaque rupture, thrombosis, and vascular occlusion [14]. The disturbance of vascular endothelial structure and function is a key link in the occurrence and development of atherosclerotic vascular diseases [19–21].

## 4. Nrf2

In 1994, researchers discovered Nrf2 in a study of beta-globin gene regulation. Nrf2, also known as NFE2L2, helps to regulate the cellular oxidative stress response. Nrf2 is classified in the Cap-n-Collar family of basic leucine zipper proteins, with 7 functional domains involved in the regulation of their stability or transcriptional activity [22]. Under basal conditions, Nrf2 binds to the Keap1/Cu13 ubiquitin ligase complex with a half-life of 10–30 minutes and is in a low activity state. When exposed to oxidative stress or other stimuli, the cysteine residues in Kelch-like epichlorohydrin-associated protein 1 (Keap1) are modified, decreasing its activity and inhibiting its binding to Nrf2. After release from the complex, Nrf2 enters the nucleus and forms a heterodimer with the small protein Maf (Nrf2-Maf) [7, 23]. Heterodimers connect to antioxidant response elements (AREs) in the initiation domain in a sequence-specific manner [24], promoting antioxidant enzyme transcription. These antioxidant genes have important antitumor, anti-inflammatory, antiapoptotic, antioxidant, and tissue protection effects [25, 26].

## 5. Role of Nrf2 in Atherosclerosis-Associated Endothelial Dysfunction

In addition to regulating vascular tone and permeability, healthy endothelial cells maintain hemostasis and coagulation, transport oxygen and nutrients to tissues, coordinate inflammatory and immune responses, and induce angiogenesis [27, 28]. Endothelial injury is a complex pathological process involving increased endothelial cell activation and endothelial dysfunction. When endothelial injury occurs, endothelial cells within the vascular lumen transform into a proinflammatory, proadhesive, procoagulant phenotype, in a process called endothelial cell activation. This is immediately followed by decreased endothelial NO bioavailability, altered vascular tone, and endothelial cell transformation to other phenotypes, collectively referred to as endothelial dysfunction [29–31]. Many studies have shown that Nrf2 has anti-inflammatory, proangiogenic, antioxidative damage, and mitochondrial protection roles in atherosclerosis-related endothelial cell dysfunction [32–35]. However, the mechanism of Nrf2 in endothelial cell oxidative stress and mitochondrial damage is unclear. Therefore, the role of Nrf2 in oxidative stress and mitochondrial injury in endothelial cells will be elaborated.

## 6. Nrf2 Improves Endothelial Dysfunction by Inhibiting Oxidative Stress

### 6.1. Oxidative Stress and Endothelial Dysfunction

Oxidative stress is important in mediating cytokine production and secretion, linking ROS to endothelial dysfunction [31, 36]. NO is the main reason that endothelial cells maintain vascular homeostasis. The production of NO in vivo uses l-arginine as a substrate, instigated by NO synthase (NOS), and generates l-citrulline and NO [37]. Several factors affect the vascular distribution of NO ultimately resulting in reduced NO release, including impaired endothelial cell membrane receptors paired with NO agonists or reduced endothelial diffusivity, physiological changes, inappropriate use of l-arginine, reduced enzymes responsible for converting or changing cGMP levels, reduced substrates for synthesizing NO, or significant degradation of NO [38]. Endothelial dysfunction is associated with decreased NO bioavailability as a result of decreased NO production and increased consumption. Superoxide anions react with NO to generate peroxynitrite (ONOO-), which promotes protein tyrosine nitration in vivo, affects protein structure and function, and further impairs endothelial function [39, 40]. Guzik et al. [41] studied the role of superoxide production due to NAD(P) H oxidase in human atherosclerosis in relation to NO-mediated vasodilation. Xu et al. [42] found that berberine protects against the human coronary artery endothelial cell disorder induced by Kawasaki disease by impairing oxidation and endoplasmic reticulum stress.

### 6.2. Nrf2 Improves Endothelial Dysfunction by Inhibiting Oxidative Stress

A growing body of evidence suggests that the Nrf2-driven antioxidative pathway has vascular protective effects in CVDs such as atherosclerosis, hypertension, diabetes, myocardial infarction, and heart failure [43, 44]. Oxidative damage induced by ROS or lipid peroxidase aggravates endothelial cell damage, which in turn activates the transcription factor Nrf2 in endothelial cells, and stimulated Nrf2 exerts its protective capacity by inducing downstream gene transcription [45]. This pathway involves more than 200 genes with antioxidative capabilities by increasing the ability of cells to combat oxidative stress and promote cell survival. These genes include antioxidant proteins that maintain intracellular glutathione homeostasis and decrease intracellular reactive oxygen levels, phase II detoxification enzymes, such as glutathione S-transferase (GST) and NADPH quinone oxidoreductase 1 (NQO1), which are mainly involved in decomposing toxic substances and promoting the metabolism and elimination of toxic substances and transporters, including multidrug resistance-related proteins implicated in the control of endogenous and exogenous substance output and uptake [44, 46, 47]. Chen et al. [8] discovered that expressing Nrf2 in human aortic endothelial cells increased ARE-driven transcriptional activity and increased intracellular HO-1 protein levels to protect endothelial cells from tumor necrosis factor (TNF)-*α*-mediated cytotoxicity. Ginsenoside Rg3 upregulates the Nrf2-ARE pathway by activating AKT and improves endothelial dysfunction caused by oxidative stress [48]. Similarly, paeoniflorin inhibits the tert-butyl hydroperoxide-induced overproduction of intracellular ROS and apoptosis in human umbilical vein endothelial cells via the Nrf2/HO-1 signal transduction pathway [49]. Blood flow through the vessel wall causes mechanotransduction, mainly including shear stress and tensile stress, which contributes to the maintenance of endothelial function and homeostasis. Shear stress is regarded as the most important element influencing the development of atherosclerosis [50, 51]. High unidirectional shear stress promotes endothelial Nrf2 signaling, whereas arterial regions exposed to low oscillatory shear stress are prone to atherosclerosis, in part due to reduced endothelial nitric oxide synthase expression and the attenuated antioxidant and anti-inflammatory properties of Nrf2 activation [52].

## 7. Nrf2 Suppresses Endothelial Dysfunction by Improving Mitochondrial Function

### 7.1. Mitochondrial Function and Endothelial Cell Dysfunction

Mitochondrial dysfunction contributes to increased oxidative stress in atherosclerosis, promoting inflammatory responses and lesion formation [53]. Mitochondria can also produce ROS (mtROS) under basal conditions in complexes I and III of the mitochondrial electron transport chain. Simultaneously, limited ROS production has important signaling functions, which has attracted much attention. However, various pathological stressors can induce an abnormal increase in mtROS production, which in turn leads to impaired NO synthesis in endothelial cells and the production of inflammatory cytokines, which favor atherosclerosis. Therefore, targeting mtROS may be an effective way to avoid endothelial damage and atherosclerosis [54].

Endothelial cells have a low mitochondrial content, but mitochondrial dynamics are critical to maintaining endothelial cell homeostasis under normal conditions. Several studies show that altered mitochondrial dynamics are linked to increased mtROS production and are implicated in endothelial damage and various vascular diseases [55]. Moreover, mitochondrial dynamics include both fusion and fission, and proteins involved in mitochondrial dynamics contribute to guanosine triphosphatase (GTPase) function [56], including various proteins. Mitochondrial fusion proteins 1 (MFN1) and 2 (MFN2) are proteins that regulate mitochondrial outer membrane fusion with the *N*-terminal GTPase structural domain and *C*-terminus to induce mitochondrial fusion protein oligomerization. MFN2 is also associated with mitochondrial autophagy. Optic dystrophin 1 is a protein that controls mitochondrial inner membrane fusion, ensuring the consistency of mitochondrial inner membrane structure, while also participating in mitochondrial cristae remodeling. Drp1, a member of the GTPase family that is found in the cytoplasm and is involved in fission of the mitochondrial outer membrane, is the protein that regulates mitochondrial fission [57, 58]. DRP1-mediated mitochondrial fission has been linked to endothelial dysfunction, including endothelium-dependent diastolic dysfunction, reduced microvessels, and decreased wound healing and angiogenic capacity [59–61]. Regulating Drp1 phosphorylation, inhibiting mitochondrial fission, and restoring mitochondrial morphology can protect mitochondrial function in vascular endothelial cells [62]. Protein disulfide isomerase A1 (PDIA1) is a thiol reductase of the mitochondrial fission protein Drp1. In endothelial cells, depletion of PDIA1 induces the thiolation of Drp1 at Cys644, promotes mitochondrial fragmentation and increased ROS, and impairs endothelial cell function and angiogenesis [61]. Several studies have shown that reduced MFN1 and MFN2 expression increases human umbilical vein endothelial cell injury and promotes the development of atherosclerosis [63, 64]. Retinol-binding protein 4 (RBP4) incubation inhibited mitochondrial MFN1 protein expression in human aortic endothelial cells, increased mitochondrial superoxide production, and aggravated mitochondrial damage [65].

Mitophagy is a defensive process by which the body selectively removes damaged mitochondria and is a fundamental mechanism of mitochondrial homeostasis. Mitophagy promotes mitochondrial turnover and prevents the accumulation of dysfunctional organelles. A moderate amount of mitophagy can prevent endothelial cell damage and avoid further CVD development [66].

### 7.2. Nrf2 Suppresses Endothelial Dysfunction by Improving Mitochondrial Function

There is growing evidence that Nrf2 is closely linked to mitochondrial functions, including mitochondrial antioxidant defense, mitochondrial dynamics, mitochondrial autophagy, biogenesis, and mitochondria-related intermediary metabolism [67–70]. Mitoquinone (MitoQ) is a mitochondrial-targeting antioxidant. Yang et al. [71] discovered that MitoQ intervention increased Nrf2 and HO-1 expression in high glucose-induced brain microvascular endothelial cells, while improving the mitochondrial membrane potential and decreasing mtROS generation. There is evidence that mtROS is necessary for Nrf2 activation and that the Nrf2-Keap1 complex binds directly to the outer mitochondrial membrane protein PGAM5, sensing ROS from mitochondria [72]. Nrf2 regulates mtROS homeostasis via the ARE-mediated activation of antioxidant enzymes in mitochondria, and a reduction in Nrf2/ARE activity leads to increased oxidative stress and mitochondrial dysfunction in blood vessels, resulting in endothelial damage [73]. Intracellular chloride channel 1 (CLIC1) is an oxidative stress sensor in endothelial cells. CLIC1 overexpression inhibits Nrf2 nuclear translocation, contributing to the hydrogen peroxide-induced activation of mitochondrial fission in human umbilical vein endothelial cell functional impairment [74]. Zhu et al. [34] found that Nrf2 activation inhibits Drp1-mediated mitochondrial fission, improving endothelial dysfunction. As a potent antioxidant in mitochondria, coenzyme Q10 exerts beneficial effects on mouse glomerular endothelial cells by restoring the Nrf2/ARE signaling pathway and promoting mitophagy [75].

## 8. Nrf2 and Atherosclerosis

Atherosclerosis is a chronic systemic disease characterized by lipid metabolism disorders, vascular endothelial damage, lipid deposition in the vascular wall, mononuclear-macrophage hyperplasia, and atherosclerotic plaque formation. Nrf2 depletion in macrophages leads to increased foam cell formation, increases the inflammatory phenotype, and aggravates atherosclerosis [76]. *Z*-Lig, a natural benzoquinone derivative, acts as an Nrf2 inducer and protects vascular endothelial cells from atherosclerosis caused by a high-fat diet. It reduces lipid peroxidation and increases antioxidant enzyme activity in Ldlr–/–mice [77]. Nrf2 is essential for lowering serum total cholesterol and reducing atherosclerotic plaques in an apolipoprotein *E* (ApoE) knockout animal model [78]. Nrf2 knockdown significantly increased the oxLDL-induced elevation of ROS levels, increasing the risk of CVD [79]. Nevertheless, there is some evidence that Nrf2 also exacerbates atherosclerosis. Barajas et al. [78] found that ApoE (–/–) Nrf2 (–/–) mice had reduced atherosclerotic lesions, while the occurrence of atherosclerosis was not affected in ApoE(–/–) Nrf2(–/+) mice. This is consistent with the report of Barajas et al. [78, 80]. In addition, the Nrf2 signaling pathway promotes inflammasome activation and contributes to atherosclerosis progression [81]. Further research should examine the complex role of Nrf2 in atherosclerosis to give new perspectives on the future therapeutic direction of Nrf2 in atherosclerosis.

## 9. Conclusion

Atherosclerosis is a disease that imposes a heavy burden on families, society, and the nation. Vascular endothelial injury is its main driver. In this review, we explain how the Nrf2 pathway protects endothelial cells from oxidative stress and mitochondrial dysfunction. In addition, the Nrf2-mediated transcription of antioxidant enzymes reduces endothelial cell damage, which in turn improves atherosclerosis. In this context, we believe that the Nrf2-ARE pathway could be an effective therapeutic target to reduce the occurrence and development of these diseases. However, the opposite effect seen in Nrf2-deficient animals casts doubt on whether Nrf2 activation can ameliorate atherosclerosis. Therefore, additional studies are needed to explore novel therapeutics for atherosclerosis targeting the Nrf2 signaling pathway.

## Data Availability

No data were used to support this study.
